# Condition Monitoring and Predictive Maintenance of Assets in Manufacturing Using LSTM-Autoencoders and Transformer Encoders

**DOI:** 10.3390/s24103215

**Published:** 2024-05-18

**Authors:** Xanthi Bampoula, Nikolaos Nikolakis, Kosmas Alexopoulos

**Affiliations:** Laboratory for Manufacturing Systems and Automation, Department of Mechanical Engineering and Aeronautics, University of Patras, 26504 Patras, Greece; baboula@lms.mech.upatras.gr (X.B.); nikolakis@lms.mech.upatras.gr (N.N.)

**Keywords:** deep learning, artificial intelligence, transformers, autoencoders, Long Short-Term Memory (LSTM), predictive maintenance, remaining useful life

## Abstract

The production of multivariate time-series data facilitates the continuous monitoring of production assets. The modelling approach of multivariate time series can reveal the ways in which parameters evolve as well as the influences amongst themselves. These data can be used in tandem with artificial intelligence methods to create insight on the condition of production equipment, hence potentially increasing the sustainability of existing manufacturing and production systems, by optimizing resource utilization, waste, and production downtime. In this context, a predictive maintenance method is proposed based on the combination of LSTM-Autoencoders and a Transformer encoder in order to enable the forecasting of asset failures through spatial and temporal time series. These neural networks are implemented into a software prototype. The dataset used for training and testing the models is derived from a metal processing industry case study. Ultimately, the goal is to train a remaining useful life (RUL) estimation model.

## 1. Introduction

One of the key aspects of Industry 4.0 is the integration of advanced technologies into production processes. The Internet of Things (IoT), as the key enabler of Industry 4.0, allows real-time data collection from a vast network of connected devices, sensors, and systems [1,2,3]. However, the enormous amount of digital information and data, known as Big Data (BD), generated and gathered by manufacturing Information and Communication Technology (ICT) systems usually remains underutilized [4]. Accordingly, new methods and models are needed that can truly benefit the ICT landscape and improve production processes by simple monitoring, planning, control, or even online reconfiguration of a system.

The process of examining these large and complex datasets, Big Data Analytics, can uncover hidden patterns, correlations, and other insights that are not visible to the human operator and support proactive decision making, transforming raw data into useful information and the transition from information to knowledge [5]. Big Data Analytics and data-driven techniques are becoming increasingly important for condition monitoring in various industries, including manufacturing, energy, transportation, and healthcare, revealing the actual condition of production equipment. Condition monitoring is the process of monitoring the health and performance of equipment and systems to identify potential issues and prevent failures. The goal of condition monitoring is to minimize downtime and improve overall efficiency by detecting issues before they become critical [6]. In turn, this could enable a transition from time-based preventive maintenance to predictive maintenance (PdM) or a combination of them. Performing PdM on production lines—identifying potential malfunctions in production equipment and estimating its remaining useful life (RUL)—is beneficial and important as maintenance activities can be scheduled, preventing equipment failures, minimizing downtime, and optimizing maintenance activities, leading to increased production and improved overall process performance [7,8,9,10,11]. However, taking into account the existence of a wide spectrum of artificial intelligence methods and tools, it is imperative to select an appropriate model which is capable of processing both large and complex data as well as providing accurate predictions in a fast manner. The existence of this gap is the motive of the present work, which aims to deliver a methodology that takes advantage of data analytics algorithms in the processing of data captured in production lines so as to give guidelines and detect features that can be used in PdM. As such, the combination of LSTM-Autoencoders, as a preliminary preprocessing step, and Transformer is a promising solution for addressing the above-mentioned challenges.

Additionally, the aim of this work is to propose a novel approach for fault detection and RUL prediction. Autoencoders with Long Short-Term Memory (LSTM) networks and a Transformer encoder are used to assess the operational condition of production equipment and detect anomalies that are then mapped to different RUL values. A combination of two LSTM-Autoencoder networks is proposed for classifying the current machine’s health condition based on different corresponding labels and then one Transformer encoder is used for RUL estimation. The main novelty of this approach is that a separate neural network is trained for each label, leading to better results for each case. Consequently, this method can be adjusted to several types of machines and labels. The proposed approach has been evaluated in a steel industry case based on historical maintenance record datasets. Finally, the development of a prototype method and the implementation of a software prototype have shown that the proposed method can provide information regarding the machine’s health without requiring any specialization and additional skills from the industry operators.

The structure of this work is divided into six sections. After the end of the Introduction section which presents the scope, challenges, and background of the present work, the Literature Review section follows, including key points from the literature that evaluate the performance of different data analytics algorithms and present how the topics of maintenance in manufacturing processes are tackled. After the Literature Review, this work continues with the Methods, Implementation and Case study sections, where the methodology, the actions, and the means that are needed to perform predictive maintenance in the actual case from industry are mentioned. Having created the models and extracted the features, the Case study section includes a Discussion chapter which discusses the models’ outputs and their interpretations as well as the competitive advantages. Finally, in the Conclusions section the outputs of the involved developments are summarized.

## 2. Literature Review

The condition monitoring of equipment, ensuring good functionality over the years, has become a requirement/necessity for industries [6]. Some of the key reasons are the repair downtime and the increasing cost of equipment failures, due to the high technology that is hidden in each machine and robot, and machine idling, due to repair operations leading to less productivity, out of schedule deliveries, and, consequently, dissatisfied customers [12,13]. Condition monitoring also assists the transition from the traditional, reactive, and preventive type of maintenance to the modern PdM [14,15,16]. PdM relies on AI technologies to analyze significant amounts of data as close to real time as possible, detecting potential equipment failures [17,18,19]. Data-driven approaches/methodologies are effective for PdM as ML (machine learning) models can be trained on labelled data during process failure without requiring an in-depth understanding of the underlying process [20,21]. This allows industries and machine manufacturers to leverage the vast amounts of data generated by industrial equipment, IoT devices, and edge devices to predict upcoming failures in the near future and schedule maintenance activities before they occur, extending the lifetime of the component [22,23,24]. Moreover, this kind of data-driven approach allows industries to continuously improve their predictive maintenance procedures over time by updating, upgrading, and fine-tuning their ML predictive models based on new data from the production site, improving the adaptability to any changing condition, while being sure of the performance of equipment [25,26,27]. Many different ML techniques have been explored and developed for PdM applications, as noted in sources [28,29,30,31,32,33]. The choice of technique depends directly on the application as well as on the given datasets and their characteristics [34].

Convolutional Neural Networks (CNNs) are a form of deep learning technique that has found widespread use in image and video analysis [35]. CNNs can identify complex patterns in the data that are not easily noticeable by a human operator [36,37] and are capable of managing vast amounts of data, making them suitable for industrial applications where massive amounts of sensor data are generated [38]. However, CNNs need labelled data and struggle to effectively handle complex datasets when the data are homogeneous and multi-channel [39,40]. Finally, CNNs are not well suited to handle sequences of data, as they do not have the capability to maintain information from one step of the sequence to the next, like Recurrent Neural Networks (RNNs) [41,42].

Recurrent Neural Networks (RNNs) are a type of deep learning architecture specifically optimized to handle sequential data for tasks such as natural language processing, speech recognition, and time-series forecasting [10]. With their feedback loops, RNNs are able to remember information of previous units by allowing information to pass across timeline steps [43]. Despite their strength in handling sequences, RNNs struggle to maintain long-term dependencies and may degrade in accuracy over time as the length of the input sequence increases, making them less practical for real-time predictions [44]. However, researchers have developed variants of RNNs, such as LSTM networks, that address these challenges and allow for more effective use of RNNs in PdM tasks [45].

LSTM is a type of RNN that is capable of handling the vanishing gradient problem in traditional RNNs by introducing a memory cell and gating mechanism [46]. LSTMs can retain information for long sequences and are capable of handling long-term dependencies, making them suitable for sequential data tasks such as time-series forecasting, natural language processing, and speech recognition [47,48].

Autoencoders are a type of neural network that are used for dimensionality reduction and feature learning, and they consist of two main components: an encoder that maps input data to a lower-dimensional representation, and a decoder that maps the lower-dimensional representation back to the original input data [49,50,51]. Autoencoders are relatively simple to train and implement, making them a popular choice for PdM applications. However, Autoencoders are limited to working with vector-based data, and their performance can be poor with sequential data such as time series or speech signals. This is because regular Autoencoders cannot handle the temporal dependencies inherent in sequential data. To address this limitation, LSTM-Autoencoders have been proposed, which combine the sequential processing capabilities of LSTMs with the feature learning capabilities of Autoencoders [52]

LSTM-Autoencoders are a type of Autoencoder architecture that uses LSTM networks as the encoder and decoder parts. Combining LSTM and an Autoencoder creates a powerful architecture for sequence data processing tasks, such as anomaly detection, data denoising, and feature extraction [53,54]. The Autoencoder structure enables the model to learn a compressed representation of the data, while the LSTM part allows the model to capture the time-series dependencies and long-term patterns in the data. This combination results in an efficient and effective method for analyzing sequential data [55].

**Figure 1:**
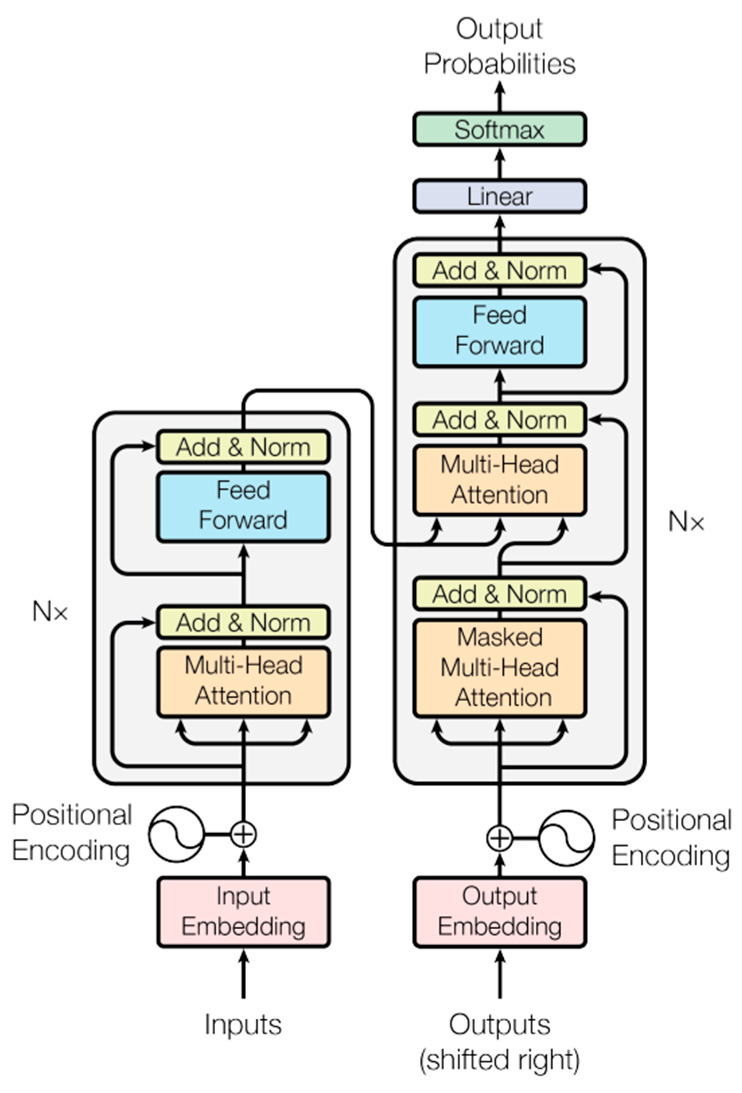
The Transformer model architecture.

Without using sequence-aligned RNNs, CNNs, or LSTMs, the Transformer is the first transduction model relying entirely on self-attention to compute representations of its input and output, becoming more and more ubiquitous in deep learning [56,57]. The Transformer architecture (Figure 1) was introduced in the 2017 paper “Attention is All You Need” [58] and has since been used in many state-of-the-art models for NLP (natural language processing) tasks such as language translation, sentiment analysis, and text classification. The main idea behind transformers is the use of self-attention mechanisms, which allow the model to focus on different parts of the input sequence and learn the relationships between them, making them well-suited for processing sequential data. Transformers eliminate the need to train neural networks with large, labelled datasets that are costly and time-consuming to produce by finding patterns between elements mathematically [59,60,61,62].

In contrast to previous approaches, the use of the attention mechanism provided by these architectures allows us to take into consideration a plethora of characteristics involved in different forms of data [63,64]. Transformers have also been used for time-series data analysis and forecasting as they are capable of capturing long-term dependencies in the time-series data [65]. The use of Transformers for that kind of data analysis has shown promising results and is an area of active research and development.

Consequently, this paper proposes and examines a supervised deep learning method, combining a set of Autoencoders with Long Short-Term Memory (LSTM) networks and a Transformer encoder, for fault detection, health condition estimation, and RUL prediction of a machine. First, the set of LSTM-Autoencoder networks classify the general current health of the machine into distinct labels, and then, only if the LSTM-Autoencoders indicate that the machine’s health is bad, one Transformer encoder is used to classify the machine’s status into specific classes corresponding to different RUL values.

## 3. Method

Currently, AI provides a plethora of tools, methods, and models for the prediction of possible equipment malfunctions. Therefore, engineers have to face the challenge of carefully selecting the most appropriate ML model. In the presented case study, alternative ML models could be implemented, e.g., GRU, which requires the use of less computational parameters, and, by extension, less computational resources, at the cost of losing long-term dependencies built up in the dataframes. The two LSTM-Autoencoders have been used as a preliminary preprocessing step in the approach in order to filter out any irrelevant information and decide if the data require further analysis from the Transformer encoder. Then, the Transformer encoder further processes and analyzes the data, mapping them into different RUL classes. So, using LSTM-Autoencoders as a preliminary preprocessing step allows a balance between computational efficiency and model performance.

### 3.1. LSTM-Autoencoders

In order to train any set of LSTM-Autoencoders, sensor data are required, derived from a production machine. After the training, the set of separate LSTM-Autoencoders can classify new sensor data that have never been seen before to different operational machine statuses. In particular, a variety of different sensors, that are placed on the machine, take measures of multiple features from the equipment and its environment. Preprocessing of the data is mandatory, as data coming from industry can be inconsistent, noisy, or even incomplete, leading to poor model performance. Apart from that, identifying the appropriate set of features associated with potential failures is a challenging task. So, in order to model the degradation process of any machine and determine the critical values, plotting the dataframe values is proposed. After the visualization of the data, and in combination with the knowledge and maintenance records of the factory specialists, related studies, and scientific dissertations of a machine, the key features can be selected.

LSTM-Autoencoders are used for the classification of the health condition of a machine to one or more categories as explained hereafter. The architecture of each LSTM-Autoencoder depends on the problem and the categories to be identified. The proposed approach requires, at a minimum, two categories to determine the health condition of the equipment: one category to represent the equipment’s good health condition, typically after maintenance or part replacement, and the other category to represent bad health conditions, such as due to degradation or failure that requires maintenance from an operational perspective. Additional categories, beyond the two mentioned, could be included based on specific needs and requirements. However, this specific study uses the minimum of two categories, namely “good health” and “bad health”, to classify the health status of the equipment. In order to classify these categories, an LSTM-Autoencoder is trained for each label, with different datasets, so the number of LSTM-Autoencoders equals the number of labels.

In order to define these different datasets and train the individual LSTM-Autoencoders, historical maintenance records are used in order to label the data based on their timestamp and the number and type of different statuses selected. Finally, a data split is performed to define, train, and test data for each LSTM-Autoencoder; 80% of the initial dataset is used for the neural network training and validation, and the remaining 20% for testing the neural network [66].

**Figure 2:**
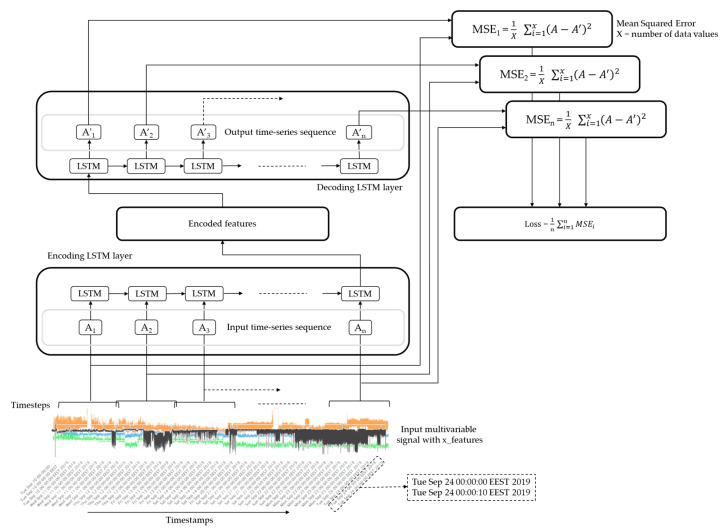
High-level LSTM-Autoencoder architecture.

Figure 2 illustrates a high-level LSTM-Autoencoder architecture. As presented in the following Equation (1), the input of each LSTM-Autoencoder is a time-series sequence, A_i_, containing the values α_ij_ of each sensor, denoting one of the variables measured at a specific time, with n being the number of features.

(1) $$A_{i}=\left[\alpha_{i1},\;\alpha_{i2},\;\alpha_{i3},\ldots ,\;\alpha_{ij}\right],\;where\;\alpha_{ij}\in R,\;with\;i,\;j\in Z\;and\;i\leq n\;\;$$

Consequently, this time-series sequence is the input of each LSTM cell of the encoder, along with the hidden output from the previous LSTM cell. Finally, the output of the encoder is a compressed representation of the input sequence, the learned representation vector, which includes all the hidden states from all the previous encoder LSTM cells. This output is fed then into the decoder to reconstruct the original input sequence, processing these encoded features through a series of LSTM decoder cells. As presented in Equation (2), the output of the decoder layer is a reconstruction of the initial input time-series sequence A′_i_, containing the reconstructed values α′_ij_ of each sensor.

(2) $$A_{i}^{\prime }=\left[\alpha_{i1}^{\prime },\;\alpha_{i2}^{\prime },\;\alpha_{i3}^{\prime },\ldots ,\;\alpha_{ij}^{\prime }\right],\;where\;\alpha_{ij}^{\prime }\in R,with\;i,\;j\in Z\;and\;i\leq n\;\;$$

After the LSTM-Autoencoder training, the model is evaluated by feeding the test data, defined earlier, as input to the model, and then, the reconstructed values are compared with the input values. The metric used to evaluate the model is the Mean Squared Error (MSE) as presented in Equation (3).

(3) $${MSE}_{i}=\frac{1}{n}\sum_{i=1}^{n}{\left(A_{i}^{\prime }-A_{i}\right)}^{2}\;\;$$

**Figure 3:**
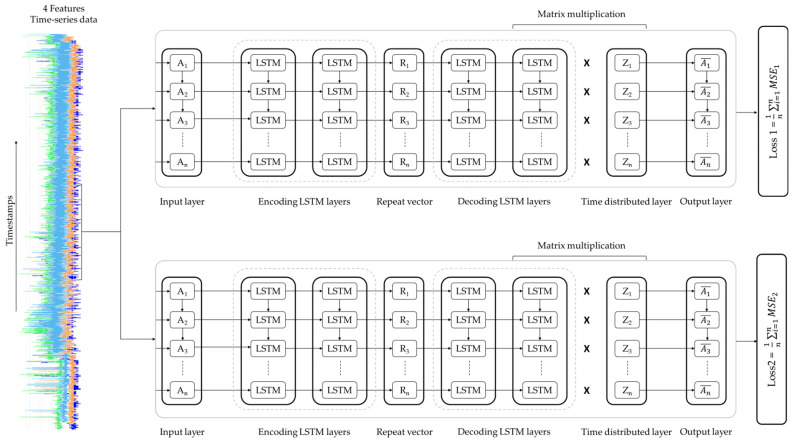
LSTM-Autoencoder architecture set.

Following the training phase, new data, that the LSTM-Autoencoders have never seen before, are provided as input to the networks, and each of them produce different reconstructed values for the same input, as depicted in Figure 3.

The integration of outputs from the two separate LSTM-Autoencoders is achieved through a decision rule, based on their reconstruction losses, compared to the input. The LSTM-Autoencoder with the lower reconstruction loss indicates better recognition of the input dataset, and consequently, the input sequence is classified into the same category state as the one used to train this specific LSTM-Autoencoder.

**Figure 4:**
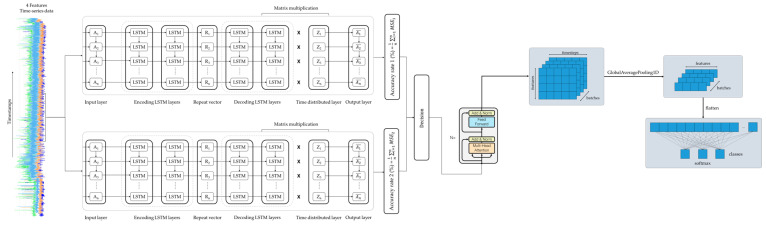
LSTM-Autoencoders and Transformer encoder integration.

In this approach, LSTM-Autoencoders serve as a preprocessing step. If the LSTM-Autoencoders classify the health status of the equipment as a “good state”, further analysis from the Transformer encoder is unnecessary. Otherwise, in case that the LSTM-Autoencoders classify the health status of the equipment as a “bad state”, the same input data are used as input to a Transformer encoder in order to identify its remaining useful life (Figure 4).

### 3.2. Transformer Encoder

The Transformer encoder is used for the identification of the current machine’s health condition and mapping it to remaining useful life (RUL) by processing and extracting meaningful information from the input data and making predictions.

In the proposed approach, three (3) classes are used for the classification representing different health states of the machine. The data that belong to Class 0 represent the health state of machines with an RUL of 3–4 days. The data that belong to Class 1 represent the health state of machines with an RUL of 2–3 days. Finally, the data that belong to Class 2 represent the health state of machines with an RUL of 1 day.

In order to label the data into the three (3) different classes, historical maintenance records are taken into consideration based on their timestamp. Finally, a data split is performed to define, train, and test data for each LSTM-Autoencoder; 80% of the initial dataset is used for the neural network training and validation, and the remaining 20% for the neural network testing.

**Figure 5:**
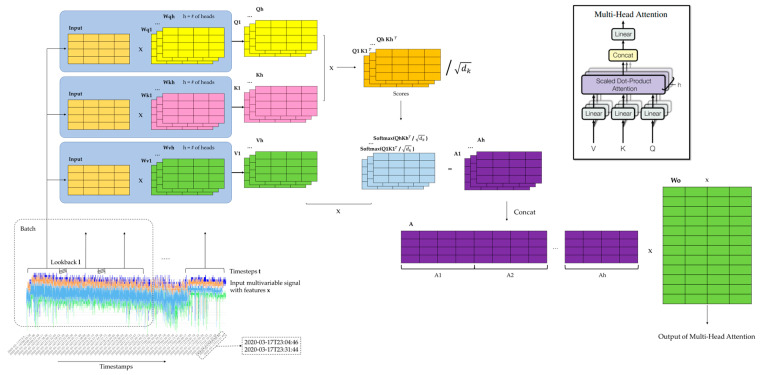
Transformer encoder Multi-Head Attention.

**Figure 6:**
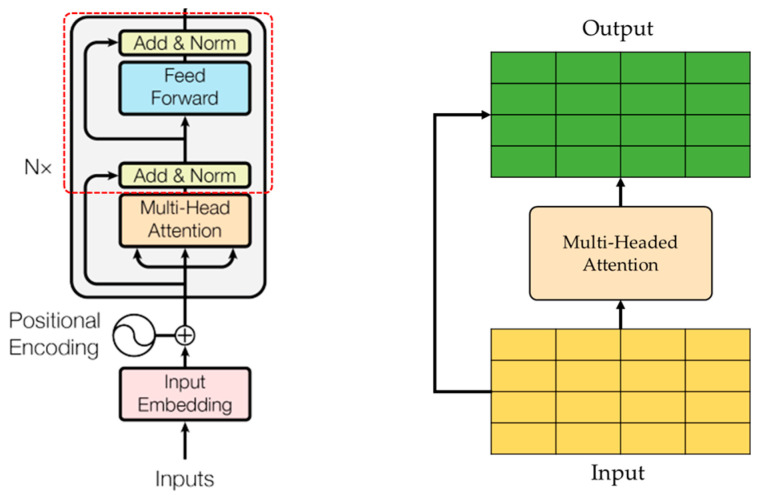
Transformer model residual connection.

Figure 5, illustrates the Transformer encoder’s Multi-Head Attention architecture. The input of the Transformer encoder is a window from time-series data that are processed independently and contain the values of each sensor. After the Q, K, and V matrixes are generated for each head independently, the next step is the matrix multiplications between the Queries matrix and the transposed Keys matrix, determining the relationships or the similarity of the Query and the Key values (the scores). These scores are then scaled down by being divided by the square root of the Query and Key dimension in order to avoid any exploding effect. SoftMax is then applied to the scaled score matrixes in order to obtain the attention weights. Finally, the attention weights of the multiple heads are multiplied with the value matrixes in order to produce one matrix for each head that contains the information of a value corresponding to the whole input. So, as the Transformer model has multiple heads (# of heads = h), the output is h matrixes. Finally, all separate h outputs from each Attention Head are concatenated and then multiplied with the Wo matrix in order to output a matrix with the same shape as the input. The output of the Multi-Head Attention is then added to the original input (Figure 6) and passes through a normalization layer, making the model more robust and stable during training.

**Figure 7:**
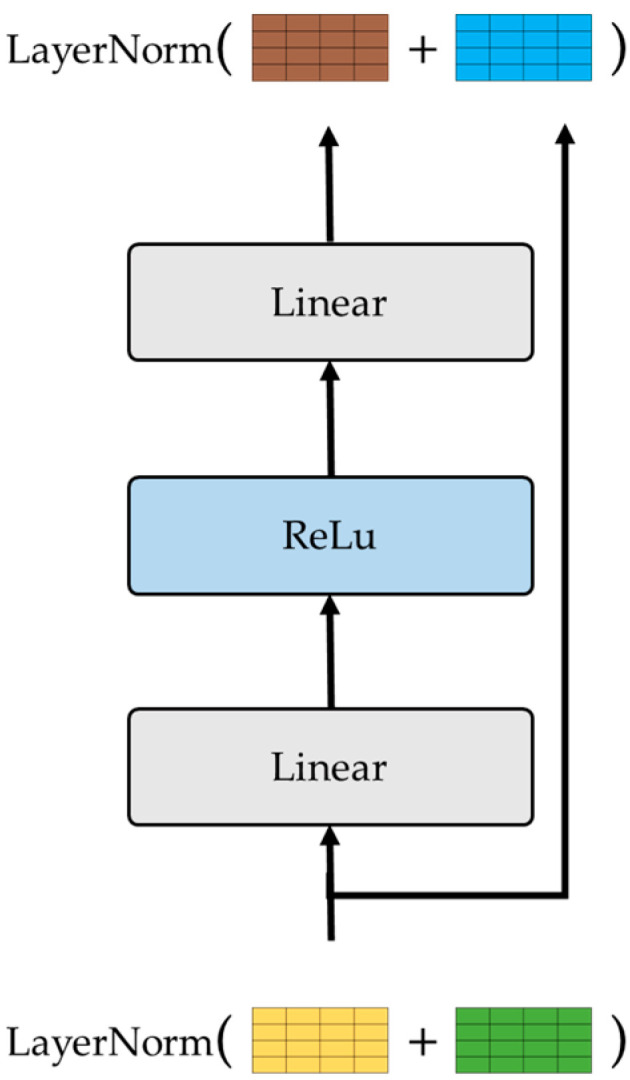
Transformer model Feed Forward network.

After the normalization, the output is then passed through a Feed Forward network (Figure 7) and the output is added to the input and normalized again. Finally, the output of the Transformer encoder is a continuous representation of the input containing all the attention information that captures all the dependencies within the sequence. The output is further processed and passes through GlobalAveragePooling1D in order to produce the final output of the model and output the probabilities of the # of classes.

After the model training, the performance of the model is evaluated through the sparse_categorical_accuracy. This metric calculates the percentage of correctly classified samples in the dataset by comparing the predicted class labels with the true class labels.

## 4. Implementation

**Figure 8:**
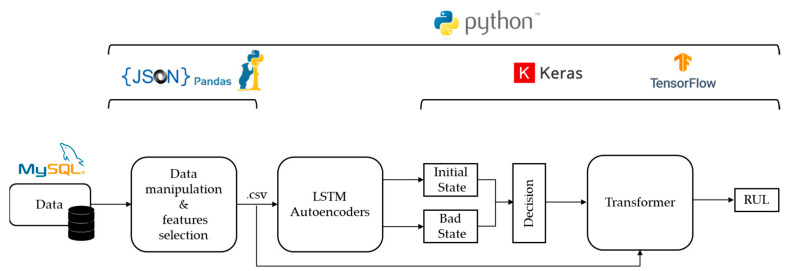
LSTM-Autoencoder and Transformer model implementation.

For the testing and the validation of the proposed approach and its potential usefulness for real-world applications, a prototype software system was implemented using Python 3.7, incorporating the aforementioned method [67]. The system was integrated using a computer with an Intel i7 processor (Intel(R) Core (TM) i7-3770 CPU @3.40 GHz 3.80 Ghz), manufactured by Intel (Santa Clara, CA, USA). In terms of processing power, the computer was equipped with an eight-gigabyte RAM memory from Samsung. Finally, the aforementioned system was hosted and tested on a computer running Microsoft Windows 10. Figure 8 illustrates a high-level representation of the LSTM-Autoencoder and Transformer network implementation.

At first, the sensor data were imported to the implemented system as JSON files, processed to remove missing values, and finally converted to a dataframe format using the Pandas library. In the final dataframe, each column represented the values of a single sensor, a feature, sorted in chronological order based on their timestamp. The selection of features, used to determine the level of degradation of the machine, was based mainly on human knowledge of the equipment and process and our bibliographic research. Finally, in order to increase the model performance, at a second level, two labels were used for the LSTM-Autoencoder network, identifying the good and bad operating condition of the monitored equipment, and then three labels were used for the Transformer network, identifying the RUL of the monitored equipment through classification.

In order to implement the LSTM-Autoencoders, the Keras library was used. Keras is a popular Python library that is widely used for developing and evaluating deep learning models as an open-source software library that provides a user-friendly interface for designing and training neural networks. In the aforementioned proposed approach, the training dataset was segmented based on historical maintenance records and then two separate LSTM-Autoencoders were trained using data corresponding to each of the two equipment states, namely good and bad. After the training the two separate LSTM-Autoencoders, newly arrived data were fed into each of the two separate LSTM-Autoencoders, which are connected in parallel, in order to classify them into one of the two supported labels, “bad state” or “good state”.

Then, in order to implement the Transformer model, Keras library was also used. In case the LSTM-Autoencoder result is that the machine is in a bad state, the Transformer model will take the same input in order to further process the data and make a classification of the RUL of the machine.

Finally, during the experimentation stage, the accuracy of the system’s results was cross-validated using the actual maintenance records provided by the use-case owner, as described in the following section.

## 5. Case Study

### 5.1. Hot Rolling Mill

**Figure 9:**
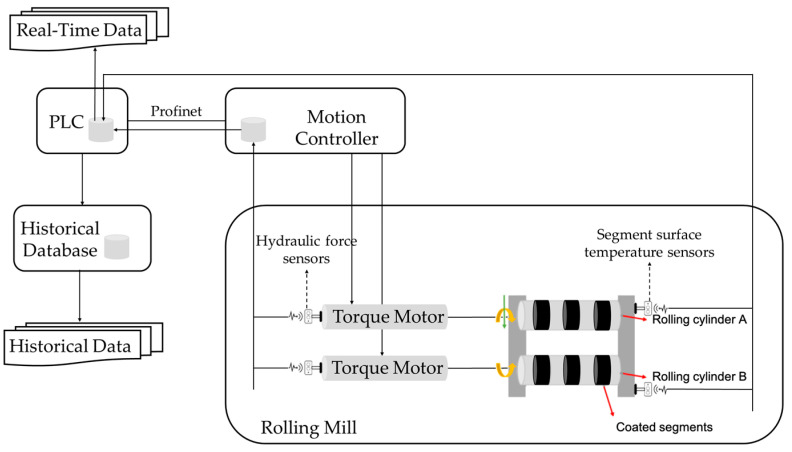
Hot rolling mill machine diagram.

The aforementioned approach was implemented into a software prototype that was trained and tested in a real-world steel production industry case. The data used in this study were derived from a hot rolling mill machine that is used for producing metal bars. Figure 9 illustrates a high-level diagram of the rolling mill machine components and their connectivity. Sensor values were initially stored in a local database on the motion controller and then transferred to a Programmable Logic Controller (PLC) database, and finally, in a historical database. Real-time data were transmitted from the PLC database to the PC for RUL prediction via communication channels. Additionally, as the developed framework was implemented on an industrial intranet, and there was no external communications/exchange of data outside the factory, no mechanisms for data privacy and security were incorporated.

The rolling cylinders of the hot rolling mill have different geometrically coated segments attached to them, which are used to form the metal bars by applying force. The rolling mill consists of three top and three bottom segments, each with a wear-resistant coating. Regarding the preventive maintenance activities that take place for this machine, the coated segments are scheduled to be replaced approximately every sixteen (16) days or sooner in case of any unexpected damage, and the replacement of the coated segments by the maintenance personnel typically lasts about two hours. The goal and objective of this study is to enable the turn from preventive maintenance into predictive maintenance by anticipating the behaviour of the segments through RUL prediction with the use of neural networks.

### 5.2. Data Preprocessing

The hot rolling mill machine condition was monitored using a variety of sensors that measured twenty-seven (27) different factors related to the equipment and its environment, and the sensor installation and operation were carried out by the industrial case provider. Of course, data coming from industry can be inconsistent, noisy, or even incomplete, leading to poor model performance. Consequently, data preprocessing is a very important step before being used for modelling and analysis [68]. All data preprocessing for this use case was implemented through a separate software module. This module receives JSON files as input. These files contain data from twenty-seven (27) sensors, and regarding the sampling rate, it was chosen by the industrial case provider, and data were collected every five milliseconds (5 ms). However, data storage took place within one-second (1 s) intervals. Since the sampling rate was too dense, entries with zero or missing values were omitted. The latter, i.e., entry omission, does not affect data consistency and quality since these data are considered sensor faults. After completion of the above-mentioned processes, data preprocessing is finalized, resulting in the creation of unified dataframe, which is ready to be used for subsequent analysis.

### 5.3. Feature Selection

Nevertheless, identifying the appropriate parameters and features that could be linked to possible equipment failures is not an easy task. In order to select the important parameters and features for our analysis, the first step in the process involved the plotting of the data. By performing the visualization of the data, critical areas in the dataframe were identified and focused on for further analysis of the dataframes. Furthermore, in order to facilitate the process of feature selection, detailed discussions with experts from the factory were performed. As such, tacit knowledge was obtained, which, by extension, enabled us to level up the dataframe from raw data to information. Finally, the dataframe was also further elaborated by combining raw data with information from historical maintenance records. According to hot-rolling-mill-machine-related studies and scientific dissertations [69], four relevant features for our approach were selected: the surface temperature of cylinders A and B and the force of cylinders A and B on trailing arm (Table 1).

### 5.4. LSTM-Autoencoder Architecture

**Figure 10:**
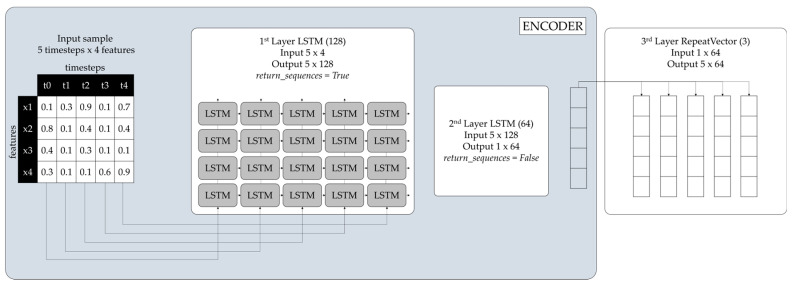
LSTM-Autoencoder encoder.

Each LSTM-Autoencoder consists of an encoder and a decoder. The number of LSTM-Autoencoder layers and neurons was selected and optimized following digital experimentation and monitoring of performance metrics. Figure 10 illustrates the architecture of each LSTM-Autoencoder and the data flow through the layers of the encoder for one sample of the dataset of size 5 × 4 (assuming that timesteps = 5).

- The input data have five timesteps and four features.
- The first encoding LSTM layer (Layer 1, LSTM(128)) reads the input data and outputs one hundred and twenty-eight (128) features with five timesteps 5 × 128, as return_sequences = True.
- The second encoding LSTM layer (Layer 2, LSTM(64)) reads the input data 5 × 128 and after reduction, outputs a vector of size sixty-four (64) 1 × 64, the encoded feature vector of the input data, as return_sequences = False.
- The repeat vector replicates the feature vector 1 × 64 five times and prepares the 2D array input for the first LSTM layer in the decoder. The repeat vector is the bridge between the encoder and decoder modules.

**Figure 11:**
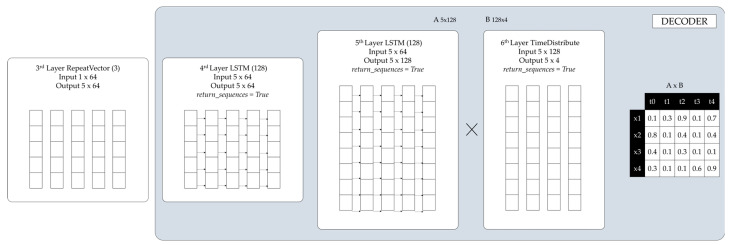
LSTM-Autoencoder decoder.

Figure 11, on the other hand, illustrates the data flow through the layers of the decoder.

- The first decoding LSTM layer (Layer 4, LSTM(64)) reads the input data 5 × 64 and outputs sixty-four (64) features with five timesteps 5 × 64, as return_sequences = True.
- The second decoding LSTM layer (Layer 5, LSTM(128)) reads the input data 5 × 64 and outputs a vector of one hundred and twenty-eight (128) features with five timesteps as *return_sequences= True*.
- The time distributed layer (Layer 6, TimeDistributed(Dense(4))) takes the output and creates 128 × 4 (number of features outputted from the previous layer × number of features) vector.
- The matrix multiplication between the output of Layer 5, 5 × 128, and the output of Layer 6, 128 × 4, resulted in a 5 × 4 output (the input and output dimensions match).

Table 2 presents the architecture of each LSTM-Autoencoder, which includes the layers of the network created, the number of parameters (weights and biases) of each layer, and the total parameters of the model, as also described previously. In machine learning and neural networks, the number of parameters in a neural network can have an impact on the processing complexity of the model [70]. In this approach, the number of trainable parameters in each network was 249.860, which resulted in the good performance of the model.

### 5.5. LSTM-Autoencoder Training and Testing

Apart from monitoring the equipment condition and data collection from the sensors, another very important piece of information is the historical maintenance records. In the aforementioned approach, two separate LSTM-Autoencoders were trained in order to classify data into one of the two supported labels, “bad state” or “good state”. Each of these two LSTM-Autoencoders were trained with a different dataset representing the different situations of the machine, defined according to the previous segment’s exchange records (Table 3).

**Figure 12:**
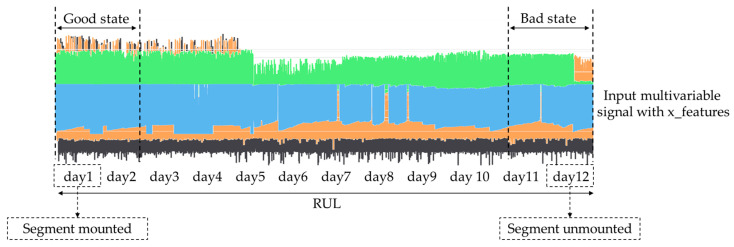
Data selection for training LSTM-Autoencoders.

As mentioned before, the coated segments are scheduled to be replaced approximately every sixteen (16) days or sooner in case of any unexpected damage and failure. So, as illustrated in Figure 12, we can assume that in the first two days that the coating was mounted, the sensor data corresponded to a machines’ good state, and vice versa: the last two days before the coating was unmounted, the sensor data corresponded to a machines’ bad state (Table 4).

Each dataset consisted of approximately 200,000 values. The datasets were then split into training and test data, with 80% of the first part of the dataset used for training and the remaining 20% used for testing. Both the training and test data were normalized to a range from 0 to 1 to facilitate faster and better training of the neural networks.

Table 5 presents the training loss results after performing multiple experiments in order to identify the ideal number of epochs, the window size, and the batch size in this use case. Epoch refers to the number of times the entire training dataset is passed through the neural network during the training process. In each epoch, the neural network goes through all the training examples in the dataset. The batch size refers to the number of samples that are processed at each training iteration, and the weights of the neural network are updated after processing each batch.

After the training of the LSTM-Autoencoders, new datasets that the two separate LSMT-Autoencoders had never seen before were then input. Each dataset was the input for both LSTM-Autoencoders and each of them produced different reconstructed values for the same input. The reconstructed values that presented a smaller reconstructed error with the input are probably recognized better by this LSTM-Autoencoder. As a result, the input dataset belongs to the same category state as the dataset that the LSTM-Autoencoder was trained with. In Table 6, the first column refers to the actual states of the monitored equipment on specific days according to the historical maintenance records of the hot rolling mill, while the last two columns present the loss generated by each one of the two LSTM-Autoencoders for the corresponding days.

### 5.6. Transformer Encoder Architecture

**Figure 13:**
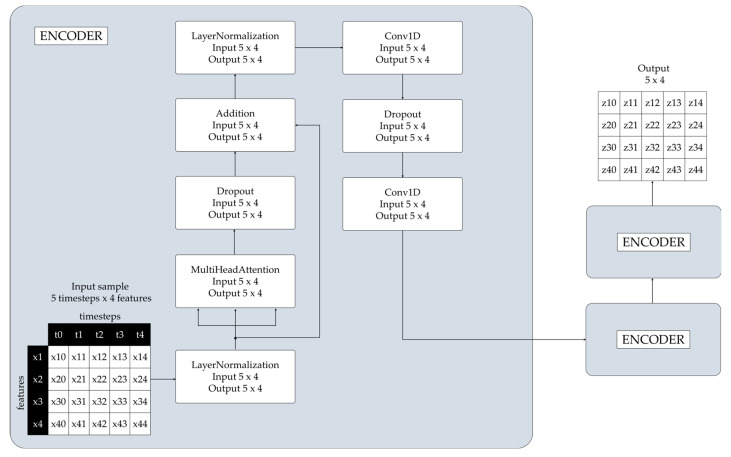
Transformer encoder.

Figure 13 illustrates the architecture of one of the Transformer encoders and the data flow through the layers of the encoder. Transformers consist of a fixed number of stacked layers [71]. After windowing, the sample input data consists of five timesteps and four features.

- A LayerNormalization layer normalizes the input data and outputs four features with five timesteps (5 × 4).
- A MultiHeadAttention layer outputs four features with five timesteps (5 × 4).
- A Dropout layer outputs four features with five timesteps (5 × 4).
- An Addition layer outputs four features with five timesteps (5 × 4).
- A LayerNormalization layer normalizes the input data and outputs four features with five timesteps (5 × 4).
- A Conv1D layer operates as a feature extractor and captures patterns, applying a 1D convolution operation to the input, and outputs four features with five timesteps (5 × 4).
- A Dropout layer randomly sets a fraction of input units to zero and outputs four features with five timesteps (5 × 4).
- A Conv1D layer applies a 1D convolution operation to the input and outputs four features with five timesteps (5 × 4).

**Figure 14:**
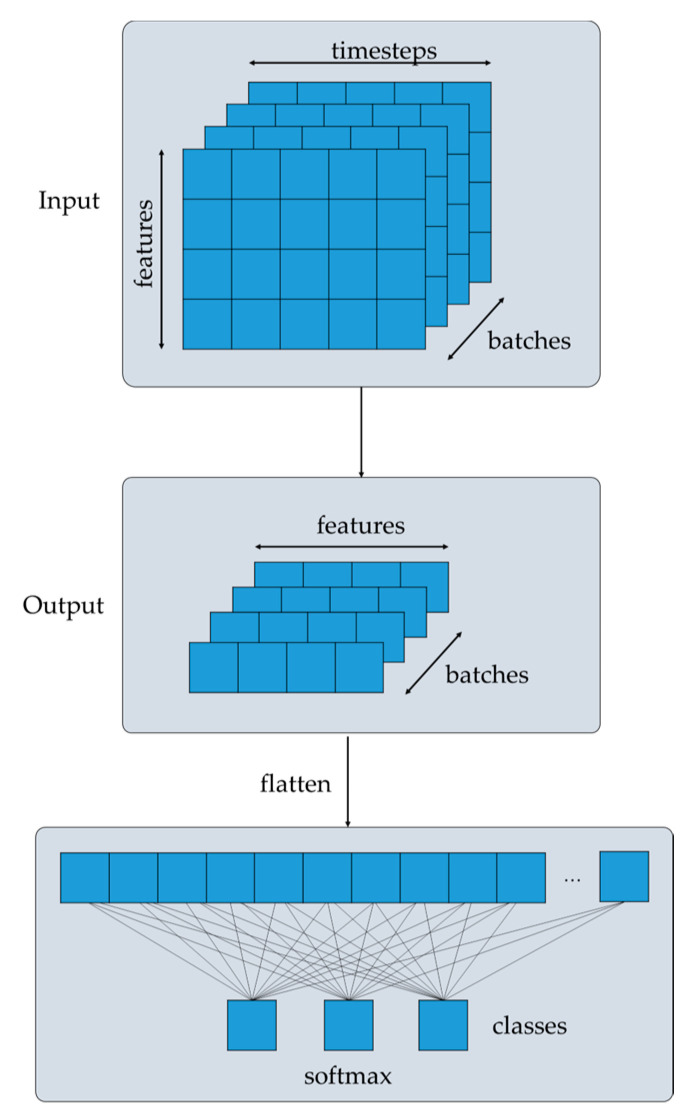
Probability generation for the classification.

Finally, after the input passes through all of the stacked Transformer encoders, the output is an encoded representation of the input. The number of stacked Transformer encoders is selected and optimized following digital experimentation and monitoring of performance metrics. The Transformer encoders create a continuous representation of the input with attention information, capturing all the dependencies within the sequence. Then, the output is further processed in order to produce the final output of the model, as depicted in Figure 14. A GlobalAveragePooling1D layer takes the input tensor and computes the average value along the timesteps of the input tensor and outputs a tensor with shape (# of samples, # of features). Then, this output is passed through the Dense layer that applies linear transformation, followed by the ReLu activation function. Then, the output of the Dense layer passes through a Dropout layer. Finally, the output of the Dropout layer is passed through a Dense layer with units = # of classes applying linear transformation followed by the SoftMax activation function. This function outputs the probabilities of the # of classes.

### 5.7. Transformer Encoder Training and Testing

**Figure 15:**
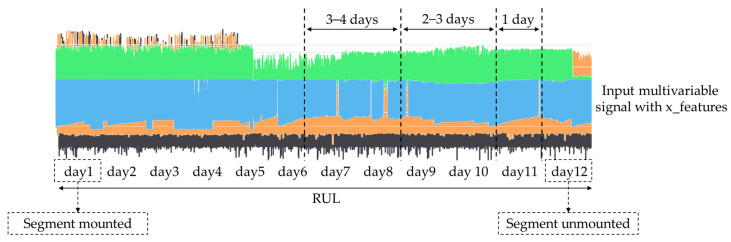
Data selection for training the Transformer encoder.

For the Transformer model training, the segment’s exchange records (Table 3) are used to label the data into different classes. For example, as illustrated in Figure 15, assuming that the new segment was mounted on day one (1) and was unmounted because of a break down on day twelve (12), the data from day 7 and day 8 can be labelled as Class 0, the data from day 9 and day 10 can be labelled as Class 1, and finally, the data from day 11 can be labelled as Class 2.

The input dataset consisted of approximately 300,000 values. The datasets were then split into training and test data, with 80% of the first part of the dataset used for training and the remaining 20% used for testing. Both the training and test data were normalized to a range from 0 to 1 to facilitate faster and better training of the neural networks.

Table 7 presents the best accuracy rate after performing multiple experiments in order to identify the ideal window size and batch size in this use case.

Following the completion of the model training phase, a series of digital experiments were conducted. For these experiments, new datasets were used, derived from the splitting of the initial dataframe. These experiments share the same methodology, yet with different datasets as input to the Transformer model. The output of each experiment is a set of classification metric values and confusion matrices over the different classes. Finally, the results from the experiments were cross-validated using the actual maintenance records provided by the use-case owner for the evaluation of the system’s performance. Each class corresponds to a different health state of the machine (Table 8).

**Figure 16:**
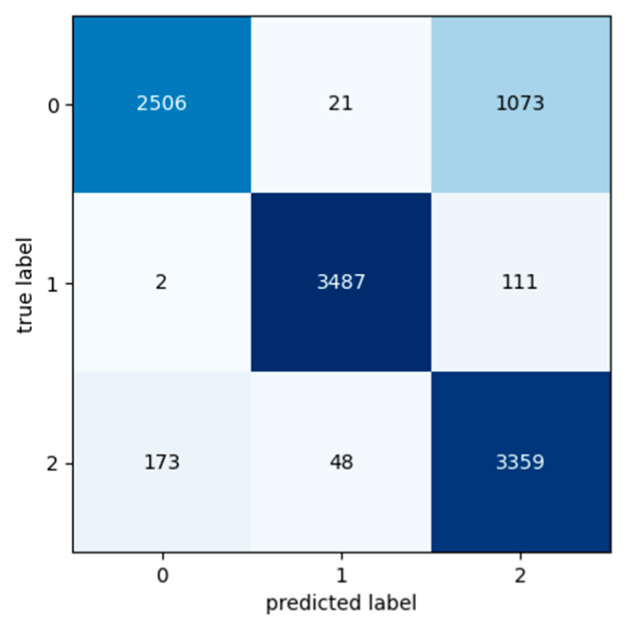
Confusion matrix: Experiment 1—maintenance because of break down.

**Figure 17:**
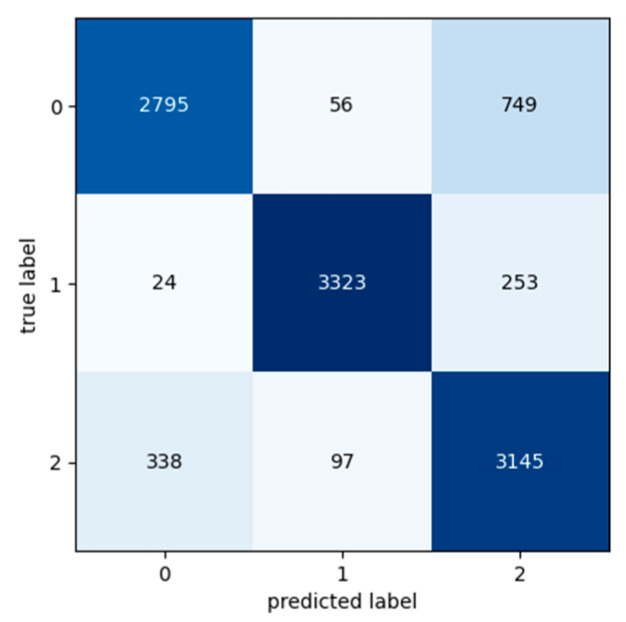
Confusion matrix: Experiment 2—maintenance because of break down.

**Figure 18:**
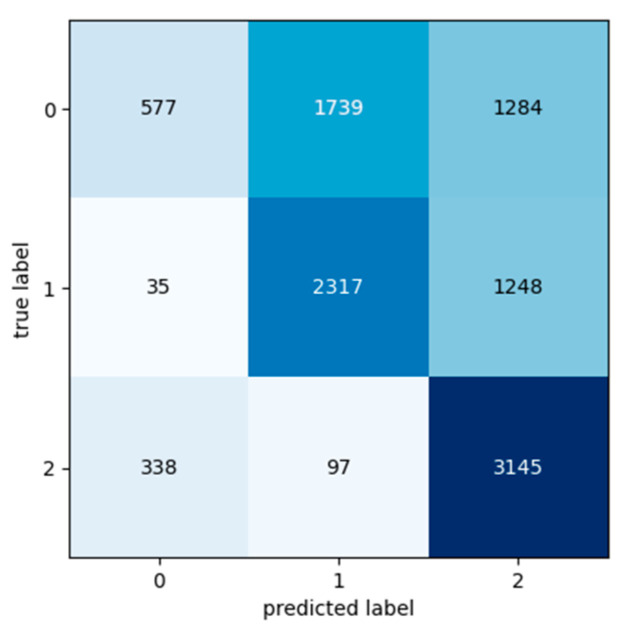
Confusion matrix: Experiment 3—maintenance because of break down.

Table 9, Table 10 and Table 11 present the classification metric values in order to evaluate the performance of the Transformer model. The metrics used for the evaluation are Precision, Recall, F1 Score and Accuracy and are calculated for each class in each input dataset. The input datasets used for the experiments were labelled as Class 0, Class 1, and Class 2 based on the segment’s exchange records. Confusion matrixes are used in order to provide a representation of the Transformer model’s actual class labels and the predictions for each class (Figure 16, Figure 17 and Figure 18). Each row of the confusion matrix represents the number of data values that belong in the real class, and each column represents the number of data values in the predicted class.

The input datasets used for the following three experiments were labelled as Class 2 despite the fact that these data were taken the day before the preventive maintenance activities based on the segment’s exchange records. As the segment exchange took place preventively and not because of a segment break down, it indicates that the machine may have had a few more days of expected life. Consequently, it is interesting to observe the Transformer model’s predictions for these cases (Table 12, Table 13 and Table 14).

**Figure 19:**
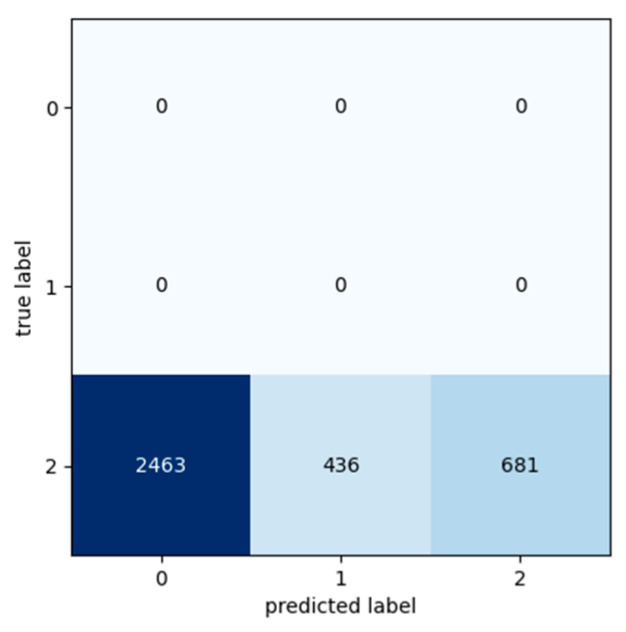
Confusion matrix: Experiment 4—preventive maintenance.

**Figure 20:**
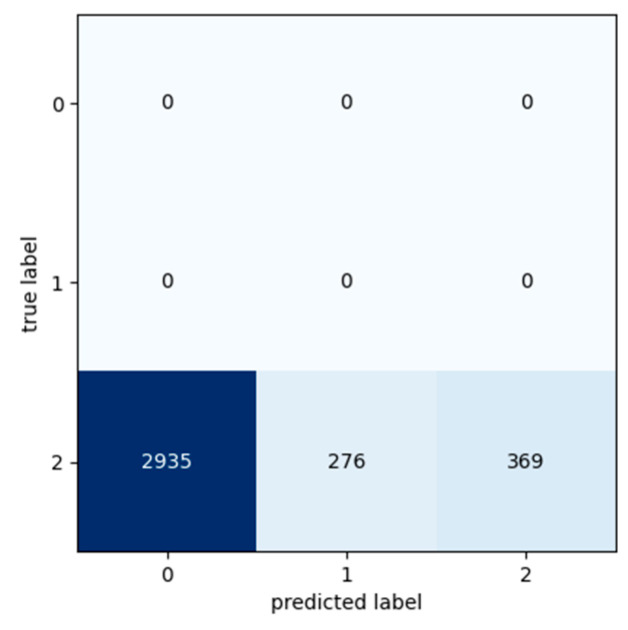
Confusion matrix: Experiment 5—preventive maintenance.

**Figure 21:**
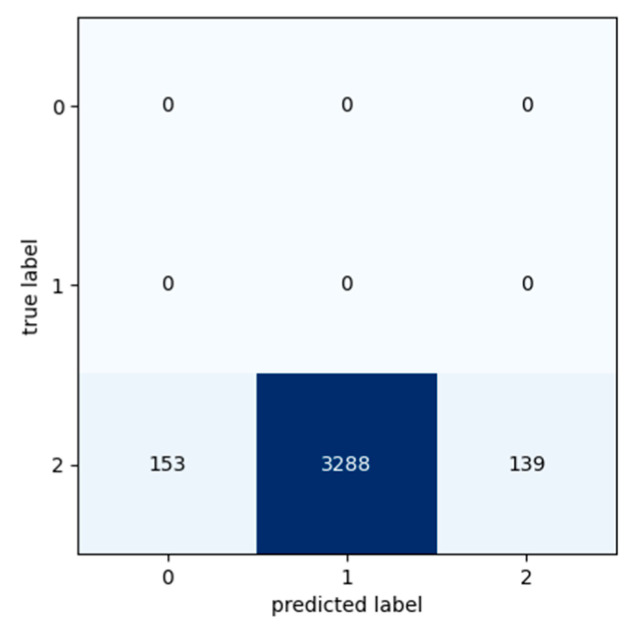
Confusion matrix: Experiment 6—preventive maintenance.

Confusion matrixes show that despite the fact that these data were taken the day before the preventive maintenance activities and should belong in Class 2, the machine may have had a few more days of expected life. According to Figure 19 and Figure 20, the Transformer model predicted that these data belong to Class 0 and have about 3–4 more days of life, while, according to Figure 21, the Transformer model predicted that these data belong to Class 1 and have about 2–3 more days of life.

### 5.8. Discussion

In order to evaluate the performance of the proposed approach, four months of machine operation data were used, and the datasets for training and testing were created based on the historical maintenance records from the hot rolling mill machine.

For the LSTM-Autoencoder (Table 6) the difference between the losses of the two LSTM-Autoencoders was enough in order to categorize and label the input data and identify the health status of the hot rolling mill machine. The datasets for training and testing created based on the historical maintenance records from the hot rolling mill machine

The results from the experiments were cross-validated using the actual maintenance records provided by the use-case owner for the evaluation of the system’s performance. According to the data presented in Table 9, Table 10 and Table 11, the Transformer model can predict the equipment’s health state, predict the remaining useful life, and prevent any failure or break down with high confidence. Additionally, the network results in Table 12, Table 13 and Table 14 show that the equipment was still in a healthy state at the time of preventive maintenance activities. Consequently, in a period of one (1) year, as preventive maintenance activities take place every sixteen (16) days, the equipment could gain (on average) approximately fifty-seven (57) more days of life and a 17,39% reduction in preventive stoppages.

As indicated in the LSTM-Autoencoder Training and Testing paragraph, the developed framework can predict the equipment’s health status and the corresponding RUL values with a high confidence rate. However, the fact that the confidence level remains less than 100% indicates that the developed framework is a complementary tool and provides good estimates for the technician/engineer, and that human intervention is still required in order to ensure seamless operation of the production line. Concretely, the developed framework can be used as a smart suggestion system which monitors the status of the equipment and interprets data, in an attempt to inform technicians/engineers whether or not the specific equipment requires maintenance to be carried out.

## 6. Conclusions

In conclusion, this study proposes a new approach for fault detection by evaluating the condition of production assets and predicting their remaining useful life (RUL). In order to integrate this solution, Autoencoders with Long Short-Term Memory (LSTM) networks were combined with a Transformer encoder to evaluate the functional status of a hot rolling mill machine in manufacturing, identify any anomalies, and map them to RUL values. Initially, a combination of two LSTM-Autoencoder networks was trained for the classification of the current machine’s health condition to the two different corresponding labels of the machine, good state and bad state. Then, a Transformer encoder was trained in order to estimate and predict the remaining useful life of this machine. The proposed method was evaluated on a hot rolling milling machine.

The novelty of the proposed approach is that in the first phase, a separate LSTM-Autoencoder is trained for one label, leading to better results, and making it easily adjustable to many labels following the exact same logic and procedure. The two LSTM-Autoencoders were used as a preliminary preprocessing step in the approach in order to filter out any irrelevant information and decide if the data required further analysis from the Transformer encoder. Then, the Transformer encoder further processes and analyzes the data, mapping them into different RUL classes. So, using LSTM-Autoencoders as a preliminary preprocessing step allows a balance between computational efficiency and model performance. Furthermore, considering the architectural characteristics of the Transformers, key elements such as non-sequential processing and self-attention mechanisms enable such models to process large datasets in real time and provide faster responses in comparison to other similar models.

Real-world data from a hot rolling milling machine were used both for training and testing of the neural networks, and the obtained results were satisfactory as presented in this study. However, during the development of the presented method, several challenges emerged. One of the key limitations was the extensive data preprocessing required. Concretely, a manual labelling process was mandatory, which was encountered by combining the dataframe with labels derived from historical maintenance records. Another key limitation was the increased complexity of the data, which was addressed by iteratively fine-tuning the hyperparameters of the model. By extension, additional experiments are necessary to be conducted using a more extensive dataset of higher data quality for a longer time period.

The results from all the different experiments show that the proposed approach is promising and can help to improve maintenance planning, reducing redundant and preventive stoppages in the production line, preventing any serious failure of the machine before it happens, and leading to a decrease in the cost of maintenance operations. Finally, the proposed method can provide information regarding the machine’s health without requiring any specialization and additional skills from the industry operators.

However, one limitation of the proposed approach arises when dealing with data of higher resolution with multiple labels, requiring multiple neural networks to identify the machine’s status. Such cases can be computationally complex, and neural networks may not be able to accurately recognize the neighbour states. Also, another limitation of this approach is the requirement for maintenance records used to label the datasets, such as component break downs and failures. These kinds of data are limited in the industry as preventive maintenance activities are planned in order to avoid this kind of critical failure of the equipment.

A next step for this approach is performance optimization by choosing different sets of hyperparameters for each network, conducting experiments, and comparing the results. Also, the robustness of the model to anomalies and noise data will be evaluated. The same approach could also be tested with more than four features and high-dimensional data, or completely different set of features for training. This expansion will allow the model to find and uncover more hidden patterns, relationships, correlations, and other insights that may remain undiscovered within the constraints of the current implementation.

Future work will also focus on evaluating the proposed concept against other machine learning methods combining different neural networks for each step, using different datasets from different real-world scenarios. In terms of implementation, and in order to minimize the framework’s response time (i.e., real-time), a better network infrastructure needs to be implemented in order to reduce network latency and system response. Furthermore, regarding the neural network operation, the utilization of high-power GPUs could further reduce prediction time. Finally, in an attempt to improve the impact of the proposed method, future work will involve the comparison of the developed model versus other statistical models, e.g., the exponential degradation model. Finally, different architectures for varying conditions will also be investigated and compared against the current approach.

## Figures and Tables

**Table 1:** Features selected.

Feature Name	Feature Value	Feature Description
Cylinder A segment surface temperature	Celsius (°C)	Surface temperature of cylinder A
Cylinder B segment surface temperature	Celsius (°C)	Surface temperature of cylinder B
Cylinder A hydraulic force	Kilonewton (kN)	Force of cylinder A on trailing arm
Cylinder B hydraulic force	Kilonewton (kN)	Force of cylinder B on trailing arm

**Table 2:** LSTM-Autoencoder: number of trainable parameters.

Layer	Type	Output Shape (Timesteps × Features)	Parameters
input1	InputLayer	5 × 4	0
lstm1	LSTM	5 × 128	68,096
lstm2	LSTM	1 × 64	49,408
repeatvector1	RepeatVector	5 × 64	0
lstm3	LSTM	5 × 64	33,024
lstm4	LSTM	5 × 128	98,816
timedistributed1	TimeDistributed	5 × 4	516
Total parameters:			249,860
Trainable parameters:			249,860
Non-trainable parameters:			0

**Table 3:** Historical maintenance records.

#	Mounted	Unmounted	RUL	Remark
1	day 1	day 12	12 days	Large piece broken out of surface
2	day 1	day 15	15 days	Large piece broken out of surface
3	day 1	day 16	16 days	Preventive maintenance
4	day 1	day 15	15 days	Large piece broken out of surface

**Table 4:** Data selected for training LSTM-Autoencoders.

#	RUL	Remark	Good Data	Bad Data
1	12 days	Large piece broken out of surface	day 1–day 2	day 11–day 12
2	15 days	Large piece broken out of surface	day 1–day 2	day 14–day 15
3	16 days	Preventive maintenance	day 1–day 2	-
4	15 days	Large piece broken out of surface	day 1–day 2	day 14–day 15

**Table 5:** LSTM-Autoencoder training loss results (%).

Loss	Window Size 5		Window Size 10		Window Size 20	
	Batch 32	Batch 64	Batch 32	Batch 64	Batch 32	Batch 64
Good State	0.0016	0.0015	0.0156	0.0224	0.0345	0.0338
Bad State	0.0071	0.0071	0.0219	0.0438	0.0630	0.0416

**Table 6:** LSTM-Autoencoder test results.

Historical Maintenance Records			Loss	
Equipment State	RUL	Input Date	AE Good State	AE Bad State
Good State	15 days	day 2	0.006	0.035
Good State	16 days	day 1	0.006	0.015
Good State	16 days	day 2	0.01	0.030
Bad State	15 days	day 14	0.037	0.005
Bad State	15 days	day 14	0.036	0.004
Bad State	15 days	day 12	0.034	0.007
Bad State	15 days	day 11	0.040	0.008
Bad State	15 days	day 10	0.043	0.008
Bad State	16 days	day 15	0.017	0.001
Bad State	15 days	day 13	0.034	0.01
Bad State	15 days	day 13	0.037	0.01

**Table 7:** Transformer encoder training accuracy results (%).

	Window Size 5		Window Size 10		Window Size 20	
	Batch 32	Batch 64	Batch 32	Batch 64	Batch 32	Batch 64
Accuracy (%)	73%	66%	81%	37%	96%	93%

**Table 8:** Classes and RUL definition.

Classes	RUL
Class 0	3–4 days
Class 1	2–3 days
Class 2	1 day

**Table 9:** Transformer results: Experiment 1—maintenance because of break down.

	Precision (%)	Recall (%)	F1 Score (%)	Confidence (%)	Support
Class 0	94%	70%	80%	70%	3600
Class 1	98%	97%	98%	97%	3600
Class 2	74%	94%	83%	94%	3580
Accuracy (%)	87%				

**Table 10:** Transformer results: Experiment 2—maintenance because of break down.

	Precision (%)	Recall (%)	F1 Score (%)	Confidence (%)	Support
Class 0	89%	78%	83%	78%	3600
Class 1	96%	92%	94%	92%	3600
Class 2	76%	88%	81%	88%	3580
Accuracy (%)	86%				

**Table 11:** Transformer results: Experiment 3—maintenance because of break down.

	Precision (%)	Recall (%)	F1 Score (%)	Confidence (%)	Support
Class 0	60%	16%	25%	16%	3600
Class 1	56%	65%	60%	64%	3600
Class 2	56%	88%	68%	88%	3580
Accuracy (%)	56%				

**Table 12:** Transformer results: Experiment 4—preventive maintenance.

	Precision (%)	Recall (%)	F1 Score (%)	Confidence (%)	Support
Class 0	0	0	0		0
Class 1	0	0	0		0
Class 2	100%	19%	32%	19%	3580
Accuracy (%)	19%				

**Table 13:** Transformer results: Experiment 5—preventive maintenance.

	Precision (%)	Recall (%)	F1 Score (%)	Confidence (%)	Support
Class 0	0	0	0		0
Class 1	0	0	0		0
Class 2	100%	10%	20%	10%	3580
Accuracy (%)	10%				

**Table 14:** Transformer results: Experiment 6—preventive maintenance.

	Precision (%)	Recall (%)	F1 Score (%)	Confidence (%)	Support
Class 0	0	0	0		0
Class 1	0	0	0		0
Class 2	100%	1%	1%	1%	3580
Accuracy (%)	1%				

## Data Availability

The data presented in this study are available on request from the corresponding author due to privacy restrictions.
